# Linkage of Multiple Sclerosis and Guillain-Barre Syndrome: A Population-Based Survey in Isfahan, Iran

**DOI:** 10.1155/2012/232139

**Published:** 2012-11-04

**Authors:** Masoud Etemadifar, Peyman Roomizadeh, Seyed-Hossein Abtahi, Sepideh Sajjadi, Amin Abedini, Aryan Golabbakhsh, Mahboobeh Fereidan-Esfahani, Mojtaba Akbari

**Affiliations:** ^1^Department of Neurology, Medical School, Isfahan University of Medical Sciences, Isfahan 81744-176, Iran; ^2^Medical School, Isfahan University of Medical Sciences, Isfahan 81744-176, Iran; ^3^Medical Students' Research Center, Isfahan University of Medical Sciences, Isfahan 81744-176, Iran; ^4^S.H.A. Research Center of Neurological-Ophthalmological Sciences (SHARNOS Co.), No. 9 Boroomand, Seyed-Alikhan, Chaharbagh Abbasi, Isfahan 81448-14581, Iran

## Abstract

*Background*. Multiple Sclerosis (MS) and Guillain Barre Syndrome (GBS) are autoimmune demyelinating disorders of Central and Peripheral Nervous system, respectively. The coexistence of these two syndromes in an individual's life span is rare. *Objectives*. To inspect throughout Isfahan MS society (IMSS) records for MS cases who had history of documented GBS whether before the onset of MS or after it. *Methods*. This retrospective survey was carried out by analyzing the clinical records of 3,522 MS patients who were registered with IMSS, from April 2003 to July 2010. Eligible cases were requested to attend to IMSS for final clinical/paraclinical examinations. *Results*. Among 3,522 (2,716 women and 806 men) MS subjects, we could identify seven patients (six females and one male) with documented diagnosis of GBS. Six patients (five women and one man) had developed MS within 6.5 ± 7.0 (range: 1–16) years after being diagnosed with GBS and one (a woman) had developed GBS three years after the diagnosis of MS. *Conclusion*. It seems that the development of MS in individuals with history of GBS is more than a simple incidental event.

## 1. Introduction

The co-existence of Guillain-Barre Syndrome (GBS) and Multiple Sclerosis (MS) in an individual's life span has been rarely reported. On one hand, GBS is an autoimmune acute polyneuropathy affecting the Peripheral Nervous System (PNS) and most commonly is triggered by an antecedent infection. The most prevailing form of GBS is Acute Inflammatory Demyelinating Polyradiculoneuropathy (AIDP) [1]. The worldwide overall incidence of GBS is estimated to be 1.1–1.8 per 100,000 which is increased after the age of 50 [2]. On the other hand, MS is an immune-mediated demyelinating disease of the Central Nervous System (CNS), which is caused by the interaction of genetic and environmental components, for example, infections [3]. MS is generally known to present a variety of clinical pictures in which relapsing-remitting (RR) pattern is the most frequent. The prevalence and incidence of MS in Isfahan province of Iran has been reported as 73.3 and 9.1 per 100,000, respectively [4, 5]. Co-existence of these two distinct disorders mostly reflects the presence of demyelinating process in both CNS and PNS. The purpose of this study was to describe the clinical and paraclinical features of individuals who had experienced both aforesaid syndromes in their lives and discuss on possible relations. 

## 2. Patients and Methods

This population-based investigation was carried out in Isfahan, a central large province of Iran (between latitudes 30 and 34 degrees north of the equator and longitude 49–55 degrees). According to Iranian national census, the population of Isfahan was estimated about 4,559,256 in year 2006 and Isfahan was similar to the rest of Iran in socioeconomic proportions and demographic features.

We retrospectively aimed to inspect the computerized clinical records of MS patients who were registered with Isfahan MS Society (IMSS) from April 2003 to July 2010. IMSS is the only referral center for this specialty in Isfahan that has sought to register nearly all MS patients from all health caring sectors of the province. Indeed, all of Isfahani MS patient must join IMSS to have insurance supports and health care facilities. Moreover, all of Isfahani neurologists are requested to present their newly detected cases to IMSS. However, we cannot rule out the possibility of missing a very small number of subjects who prefer private care. Along with registration, IMSS performs history taking and collection of follow-up data for every patient and its database includes background variables, clinical records including disease pattern (RR, secondary progressive (SP), etc.), symptoms, signs and relapse history (date, duration and type), therapeutic protocols, expanded disability status scale (EDSS), and any significant complication in patients' status. The diagnostic features and details of case collection of IMSS are well characterized elsewhere [4, 5]. Until the end of July 2010, IMSS had used the 2005 revisions of McDonald's criteria for the diagnosis of MS and by that date, total Isfahan MS (TIMS) cohort exceeded 3,522 definite MS patients (2716 women and 806 men) whose demographic features are expatiated in our recent works [5]. 

Firstly, we looked over the TIMS records for cases with GBS diagnosis at any stage of their life span. This process sought to detect cases who had developed GBS whether before MS diagnosis (by searching in clinical history records) or after it (by searching in IMSS follow-up records). Cases were included only if their GBS was confirmed and documented by a qualified neurologist (pertaining to IMSS staff or not) based on Asbury criteria [6]. Eligible cases (i.e., cases with described GBS history) were requested to attend IMSS for final clinical/paraclinical examinations and providing voluntary informed consent. The study protocol was approved by institutional ethics committee.

Secondly, we raised the question whether the concurrence of MS and GBS in these individuals happened merely incidental or not. In order to examine such a question, we compared (*χ* ^2^-statistics) the prevalence of MS in the general population with the frequency of MS among an estimated population who develop GBS in a certain period of time. The size of this population was estimated by summation of GBS incidence ranges (worldwide lower and upper values, i.e., 1.1 and 1.8 per 100,000) [2] multiplied by Isfahan population in each year. These years were attributable to the duration between the dates of the earliest and the latest onset of GBS among patients who had represented GBS prior to MS. This method of analysis was chosen due to the nature of GBS. Indeed, there is a scarcity of epidemiologic data on the prevalence of GBS whether worldwide or in Iran and the majority of previous epidemiologic studies are limited to the report of disease incidence. Hence, we estimated the number of GBS patients using incidence values. The sex ratio (male : female) of GBS (1.5 : 1) [1] was taken into account to estimate sexual proportions of these patients, thus all by-sex tests were also carried out. 

Data were analyzed by SPSS, version19. Results have been reported as a mean (±SD) and number (percent). All tests were two-tailed, and a *P*-value of <0.05 was considered as significance threshold. 

## 3. Results

In our inspections throughout TIMS cohort records, we could identify seven MS patients with accompanying GBS diagnosis giving a frequency of ~0.2% (7/3522).  Table 1 summarizes the clinical/paraclinical features of our cases. Six cases (No. 1–5, 7) were females and case no. 6 was male. The mean (±SD) ages at onset of MS and GBS were 23.8 ± 6.5 (Range: 18–35) and 18.7 ± 7.8 (Range: 3–27) years, respectively. Two cases (two males) with the diagnosis of chronic inflammatory demyelinating polyradiculoneuropathy (CIDP) were also detected in the IMSS records, which were not included in this research.

One patient (No. 7) had developed GBS three years after the diagnosis of MS. Thus, the frequency of MS patients who developed GBS in their disease course was ~0.03% (1/3522). Six patients (Cases No. 1–6) had developed MS within mean (±SD) duration of 6.5 ± 7.0 (Range: 1–16) years after being diagnosed with GBS. Thus, the frequency of MS patients with previous GBS history was 0.17% (6/3522). These six GBS patients had developed their disease between 1989 and 2006. These dates were related to the earliest (Case No. 3) and the latest (Case No. 7) onset of GBS in these cases. We estimated the total population of Isfahani GBS patients who had presented their disease between these two dates using the upper and lower incidence ranges. The epidemiologic data (Isfahan's population in each year in addition to its sexual ratios) and the statistical details (*P* value, OR, and confidence intervals) of each analysis are brought in Table 2. The frequency of MS among the estimated GBS population was significantly greater than the prevalence of MS in the general population in total (*P* < 0.0001) and by female sex (*P* < 0.0001) comparisons (Table 2).

In history taking five cases (No. 1–3, 5, 7) had experienced an infection prior to GBS symptoms. Also, case No. 2 had developed GBS symptoms during eighth month of the pregnancy. The patterns of MS and GBS in all of the cases were RR and AIDP, respectively (Table 1).

The treatment of GBS was plasmapheresis in three cases (No. 3–5) and intravenous immunoglobulin in two (No. 1, 7) (Table 1). These cases showed excellent response to treatment. Cases No. 2 and 6 did not receive any treatment for GBS due to their minimal level of disability and rapid improvement of symptoms. In sum, it is worth noting that GBS symptoms/signs were resolved well in all of seven cases and none of them had developed weakness of respiratory muscles. Also, throughout our follow-up records, we did not observe any symptoms/signs suggestive of GBS recurrence. Family history of MS or GBS or any other significant diseases was negative in all of the cases.

## 4. Discussion

In this paper, we described seven patients who suffered from both MS and GBS during their lifespan. In our study, six subjects (Cases No. 1–6) had been diagnosed with MS after development of GBS. Our analysis may provide evidence for an increase in the development of MS following GBS.

In the best of our knowledge to date, nine patients with resembling presentations to our cases (concurrent MS and GBS diagnosis) have been described in case report studies [7–14]; among whom, only two had developed GBS preceding MS (Table 3). 

In addition to the specific literature regarding the concurrency of MS and GBS, herein, the literature about the history of concurrent CNS and PNS demyelinating diseases is noteworthy. Hasson et al., in their senior report (1958) on the peripheral nerve involvement in MS, observed slight to severe degrees of demyelination in distal segments at autopsied specimens [15]. Some observations, and Pollack et al., over the following decades were in line with the aforementioned study and yielded the suspicion for the occurrence of inflammatory demyelination of PNS in MS patients [8, 16, 17]. Tachi et al., in their case report (1985), observed the concomitancy of MS and peripheral neuropathy in a twelve-year-old girl. Their para-clinical workup revealed the process of demyelination and remyelination of the PNS concurrent with the definite course of MS [18]. Another related report was by Di Trapani et al., on two MS patients with peripheral demyelinating disease. They believed that such conditions could be indicative of “common immunopathogenetic mechanisms” [19]. More specifically in the diagnosis, the engaging association of CIDP and MS was illustrated in two cases by Falcone et al. in 2006; a report by which the authors supported the concept of distribution of the T-cell autoimmunity from the central to peripheral myelin [20]. It is to be noted that CNS and PNS demyelinating diseases share common immune-pathogenic features, for example, activation of nonspecific inflammatory cascade, which is responsible for demyelination, axonal loss, and disease progression [21, 22].

From another point of view, there are some lines of evidence indicating the CNS involvement during the definite course of PNS demyelinating syndromes. For example, Thomas et al. reported the association of chronic demyelinating neuropathy with relapsing multifocal CNS disorders in six cases; which, was supported by electro-physiologic and imaging findings [23]. Mendell et al. in another interesting MRI investigation, sought to assess the rate of CNS lesions in 16 CIDP cases; six of which were found with lesions indistinguishable from those seen in MS, and three of which with definite clinical and laboratory evidence of MS. These results led the authors to conclude that “many CIDP patients have concurrent CNS demyelination” [24]. In contrast to these findings, Feasby et al. in their corresponding brain MRI study on CIDP cases, concluded that “most patients with CIDP do not have evidence of MS” [25]. Another series of studies in this regard are electrophysiological investigations, for example, the work by Gigli et al. on CIDP cases. They found abnormal evoked potentials in visual or auditory pathways and concluded that involvement of cranial nerves and CNS sensory pathways occurs during the process of CIDP [26]. Another category of studies was performed by for example, Maier and coworkers, by which they examined autopsy GBS cases for pathological changes in the CNS and PNS. Although, they concluded that CNS is frequently affected in GBS cases, they asserted that such changes are nondemyelinating [27].

Rezania and colleagues have classified previous animal model reports on the cross-reactivity of immune responses to both CNS and PNS myelin antigens. They have characterized some instances of experimental allergic neuritis (EAN) and experimental allergic encephalomyelitis (EAE) that are models for PNS and CNS demyelination, respectively. These findings, taken together, are supporting for the occurrence of CNS demyelination following PNS demyelination in EAN animals, and provide evidence for similar immunogenic epitopes in the CNS and PNS [28]. Immunization of Lewis rats with peripheral myelin led to high titers of antibodies against CNS myelin basic protein (BP) in EAN, indicating that immunological response could spread from one part of the nervous system to the other. Such a significant finding can be explained by the notion that although peripheral and central myelin have different protein composition, they share at least one common protein antigen. Specifically, peripheral myelin P1 protein is similar to CNS myelin BP [29].

A number of studies have proposed common genetic features for MS and GBS. Vedeler et al. have suggested that certain Fc *γ* receptor (Fc*γ*R) allotypes including FC*γ*RIIA and FC*γ*RIIIB were in relation with the severity of both diseases. However, they could not show any correlation between these allotypes and the disease susceptibility [30]. Moreover, other similarities between the peripheral and the central demyelinating processes can be highlighted, for example, the M3 allele of alpha-1 anti-trypsin system found in the genotype of individuals affected by MS and GBS [31].

Previous epidemiologic studies evaluating the co-morbidity of MS and GBS have reported diverse results. Eaton et al., regardless of the priority of the onset of GBS or MS, have reported an odds ratio (OR) of 19.2 for co-morbidity of these two diseases [32]. However, this co-morbidity was not reproducible when this comparison was confined to the onset of GBS during the course of MS [33]. Conversely, Langer-Gould et al. in a population-based case control study showed that MS patients were more likely to develop GBS in their disease course. Nevertheless, they could not show that MS patients were more affected by GBS prior to MS onset [34]. Our current experience might be indicative of an increased presence of past history of GBS in MS patients. 

From another epidemiologic point of view and as a particular possible link, Epstein-Barr Virus (EBV) can be considered as an environmental risk factor for the susceptibility to a variety of diseases. Exposure to EBV is known as a potential risk factor for development of MS [3]. On the other hand, EBV is considered as one of the most important causes of antecedent infections in GBS. Tam et al. reported a strong association between EBV infection and GBS risk (OR = 20) [35]. This may call a question whether the increased co-morbidity of MS and GBS is partially originated from such a common environmental cause. However, this notion is highly speculative and needs further objective evidences.

Autoimmune diseases are well known for being difficult to diagnose. GBS and MS are neurological autoimmune diseases and MS often is preceded by a prolonged time-lag between the first symptoms of the disease to the diagnosis of MS. In our study, as we used retrospective data collection, such as that utilized in a number of similar studies, there remains the possibility that diagnostic problems might be responsible for the association between GBS and MS. It cannot be completely ruled out that patients with neurological symptoms might at first wrongly be diagnosed with GBS and then later on with a correct diagnosis of MS. However, the diagnosis of both GBS and MS cases, in our center, was based on the reputed and well-established diagnostic criteria. It is also to be noted that the mere coincidence might be responsible for the concomitancy of these two entities in a case or a number of cases. Moreover, because of the rarity of GBS, it may only take few false-positive GBS diagnoses among patients who actually were developing MS to create a strongly increased risk of MS in GBS patients. Taking together, MS and GBS are distinct entities pertaining to different parts of nervous system. Nevertheless, in line with some of previous reports, our findings might be suggestive of a linkage between the two diseases. Further population-based studies are needed to enable a field to be opened up for more accurate statistical measures in this regard.

## Figures and Tables

**Table 1:** Characteristics of seven patients with the diagnosis of both Guillain-Barre' Syndrome and Multiple Sclerosis.

Case number	1	2	3	4	5	6	7
Date of birth	1979	1974	1986	1986	1981	1982	1984
Sex	F	F	F	F	F	M	F
Age at MS onset	29	35	18	18	21	27	19
Age at GBS onset	27	19	3	17	18	25	22
Presenting symptom of MS	Facial paresthesia	ON	Right hemiparesia	ON	ON	ON	ON
Post-MS follow-up (year)	2	1	6	6	8	1	7
Final EDSS	1	1	1	1	1.5	1	1.5
Current medications of MS	Avonex	Azathioprine	Azathioprine	Avonex	Avonex	Avonex	Avonex
Probable GBS antecedent infections	Respiratory tract infection	Respiratory tract infection	Gastrointestinal infection	None	Respiratory tract infection	None	Gastrointestinal infection
GBS treatment	IVIg	None	Plasmaphresis	Plasmaphresis	Plasmaphresis	None	IVIg
Hospitalization due to GBS (day)	7	5	9	13	6	4	22

MS: Multiple sclerosis, GBS: Guillain-Barre' Syndrome, EDSS: expanded disability status scale, ON: optic neuritis, IVIg: intravenous immunoglobulin, M: male, F: female.

**Table 2:** Frequency of MS in the general population and in two estimated GBS populations; the comparison of two frequencies via χ^2^ statistics.

	Total comparison		By female sex comparison		By male sex comparison	
	Lower worldwide incidence	Upper worldwide incidence	Lower worldwide incidence	Upper worldwide incidence	Lower worldwide incidence	Upper worldwide incidence
GBS incidence rate (per 100,000)	1.1	1.8	1.1	1.8	1.1	1.8
Frequency of MS in estimated GBS patients between 1989 and 2006*	6/798	6/1305	5/319	5/522	1/479	1/783
MS in general population**	**3522/4804458**		**2718/2343459**		**804/2460999**	
*P* value	<0.0001	<0.0001	<0.0001	<0.0001	0.38	0.63
*χ* ^2^	41.25	21.56	46.07	25.03	0.75	0.23
OR (95% CI)	10.33 (4.18–23.81)	6.3 (2.55–14.5)	13.71 (5.01–34.33)	8.33 (3.05–20.78)	—	—

MS: Multiple sclerosis, GBS: Guillain-Barre' Syndrome, OR: odds Ratio, CI: confidence interval.

*The population in each year within this period are as follows (count (year)): 3511631 (1989), 3589238 (1990), 3682444 (1991), 3718789 (1992), 3768620 (1993), 3819119 (1994), 3870295 (1995), 3923255 (1996), 3982496 (1997), 4042631 (1998), 4103674 (1999), 4165639 (2000), 4228540 (2001), 4292390 (2002), 4357205 (2003), 4422998 (2004), 4489785 (2005), 4559256 (2006). (Source: Iranian Central Bureau of statistics).

**The epidemiologic data of MS in Isfahan (i.e. prevalence and sex ratio) was extracted from our recent works [5].

**Table 3:** Case report studies describing the co-existence of Guillain-Barre' Syndrome and Multiple Sclerosis.

Case report	Author(s) [Reference]	Year of report	Country	Number of patients	sex	Age at onset of GBS	Age at onset of MS	Diagnosis of MS	Type of MS	Type of GBS	Antecedent infection or surgery before GBS
1	Forrester and Lascelles [7]	1979	England	2	M	46	53	Definite	NA	NA	Probable
					M	49	32	Definite	NA	NA	Respiratory tract infection
2	Lassmann et al. [8]	1981	Austria	1	F	26	26	Definite	NA	NA	—
3	Best [9]	1985	Scotland	1	F	72	NA	Probable	NA	NA	Respiratory tract infection
4	Sanders and Lee [10]	1987	England	1	F	30	27	Definite	NA	NA	Fever and diarrhea
5	Pareyson et al. [11]	1993	Italy	1	F	33	25	Definite	NA	NA	—
6	Rio et al. [12]	1996	Spain	1	F	7, 21	26	Probable	NA	NA	Flu
7	McCabe et al. [13]	1998	USA	1	M	44	29	Definite	PR	NA	Surgery
8	Capello et al. [14]	2000	Italy	1	F	45	24	Definite	RR	AMSAN	—

GBS: Guillain-Barre' syndrome, MS: Multiple sclerosis, M: male, F: female, NA: not available, PR: progressive relapsing, RR: relapsing remitting, AMSAN: acute motor and sensory axonal neuropathy.
